# A Rare Presentation of a Transverse Mesocolic Internal Hernia: A Case Report

**DOI:** 10.7759/cureus.68765

**Published:** 2024-09-06

**Authors:** Danielle A Rowe, William B Bowers, Heather L Mateja, Eliesther F Rivera, Landry K Umbu, Pablo G Giuseppucci

**Affiliations:** 1 College of Medicine, American University of Antigua, Osbourn, ATG; 2 Department of General Surgery, American University of Antigua, Osbourn, ATG; 3 Department of Surgery, American University of Antigua, Osbourn, ATG; 4 Department of General Surgery, Western Reserve Health Education, Warren, USA; 5 Department of General Surgery, Trumbull Regional Medical Center, Warren, USA

**Keywords:** bowel obstruction, hartmann reversal, internal hernia, transmesenteric hernia, transverse mesocolon hernia

## Abstract

Internal hernias (IHs) are a rare but potentially life-threatening cause of bowel obstruction, with a high morbidity and mortality rate if not promptly diagnosed and treated. This case report highlights the clinical course of a 75-year-old female who developed a transverse mesocolic internal hernia, a subtype of transmesenteric hernia (TH), following a Hartmann reversal procedure. The patient presented to the emergency department (ED) with a sudden onset of severe, diffuse abdominal pain. Her medical history was significant for systemic lupus erythematosus, pulmonary fibrosis, multiple pulmonary embolisms, and a recent Hartmann reversal procedure the month prior. Initial imaging suggested postoperative ileus, but the patient's symptoms persisted despite conservative management. Subsequent imaging raised suspicion of an internal hernia, and on hospital day 6, an urgent diagnostic laparoscopy revealed a herniated segment of the small bowel through a defect in the transverse mesocolon with herniation into the lesser sac. The herniated bowel was successfully reduced, and the defect was repaired. The patient had an uneventful recovery and was discharged in stable condition. Transmesenteric hernias, though more common in the pediatric population, can occur in adults, particularly following abdominal surgery. Diagnosis can be challenging due to variable symptoms and imaging findings. However, prompt recognition and surgical intervention are crucial to prevent complications such as bowel ischemia and strangulation. This case underscores the importance of considering internal hernias in the differential diagnosis of small bowel obstruction (SBO), especially in patients with a history of recent abdominal surgery. Early diagnosis and timely surgical management are essential for a favorable outcome.

## Introduction

An internal hernia (IH) is defined as the protrusion of abdominal viscera, most commonly the small bowel, through a normal or an abnormal peritoneal or mesenteric aperture within the confines of the peritoneal cavity [1,2]. IHs can be congenital or acquired. Congenital hernias are present at birth and occur through weaker points in the abdominal wall [2]. Acquired hernias are caused by trauma, surgical procedures, or other pathological conditions [2,3]. Acquired internal hernias are particularly linked to bariatric surgery due to the creation of the roux loop [3]. This has caused an increase in the presentation of transmesocolic hernia, transmesenteric hernia (TH), and retroanastomotic hernia [2]. IH can be classified based on location, with the main types being paraduodenal (53%), pericecal (13%), foramen of Winslow (8%), transmesenteric and transmesocolic (8%), intersigmoid (6%), and retroanastomotic (5%), with the overall incidence of IHs being 0.2%-0.9% [2]. Though the incidence of IHs is minimal, the morbidity and mortality, when they do occur, are as high as 50% [2]. Swift diagnosis and often operative management may be required. Here, we present the case of a 75-year-old female patient who presented with sudden-onset abdominal pain and was subsequently diagnosed with transverse mesocolic IH, which required operative management. We write this article to bring awareness to this rare but life-threatening disease in hopes of leading to swifter diagnosis and management.

## Case presentation

A 75-year-old female with a significant medical history including systemic lupus erythematosus, pulmonary fibrosis, multiple pulmonary embolisms, hypertension, hyperlipidemia, hypothyroidism, and diverticulitis presented to the emergency department (ED) with sudden onset of severe, diffuse abdominal pain. The patient was well-known to the surgical team, having undergone a robotic-assisted laparoscopic Hartmann procedure one year prior for perforated diverticulitis, followed by a successful, uneventful Hartmann reversal one month prior to this presentation. She reported a stable recovery post reversal until this episode, denying associated symptoms such as nausea, vomiting, fever, chills, changes in bowel habits, or hematochezia.

**Figure 1:**
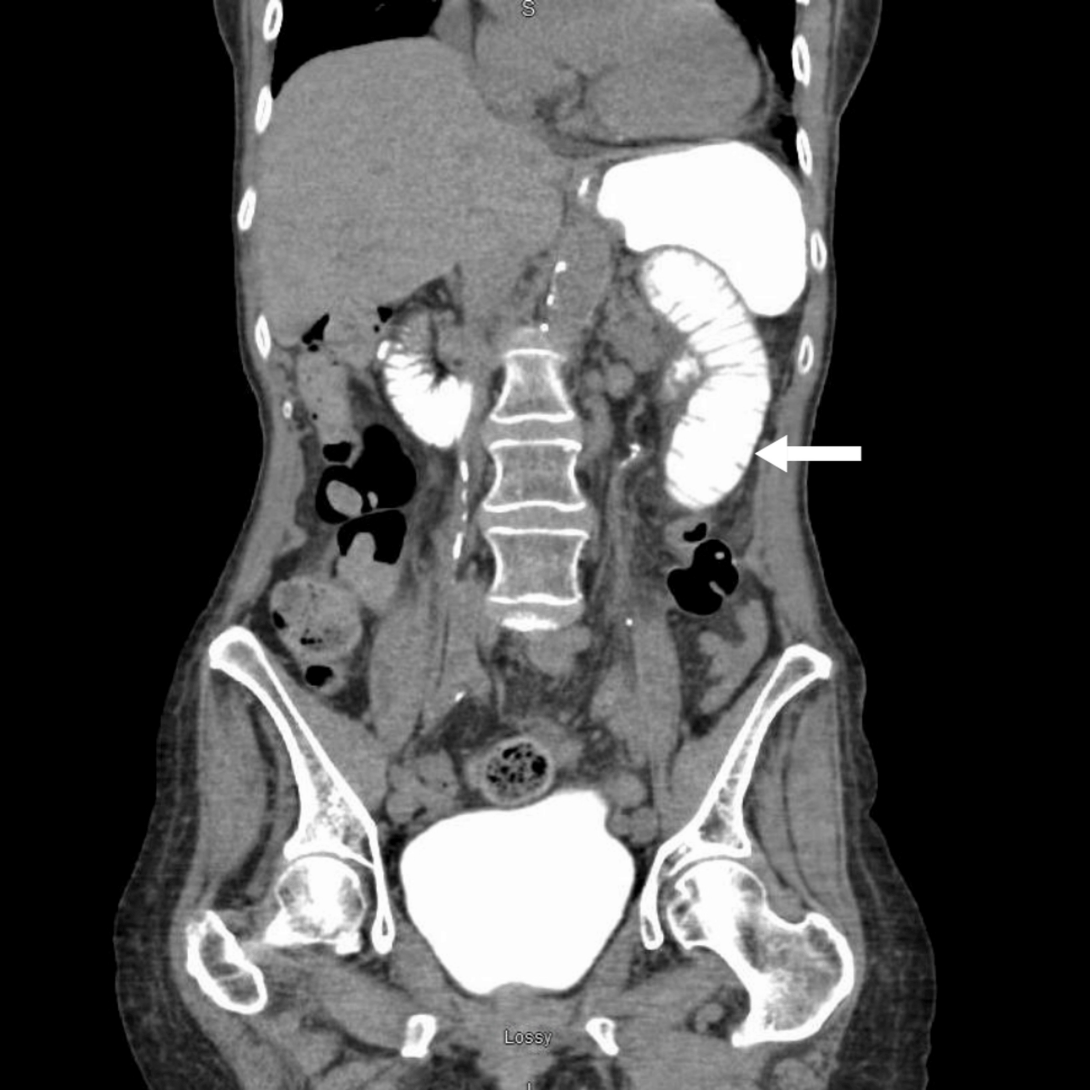
Coronal view of a contrast-enhanced computed tomography (CT) scan of the abdomen and pelvis (with intravenous and oral contrast). Demonstrating findings concerning small bowel obstruction versus ileus, with contrast reaching the proximal small bowel (white arrow).

In the ED, the physical examination revealed diffuse abdominal tenderness to palpation, with well-healed postsurgical wounds exhibiting no signs of erythema, warmth, drainage, or purulence. The patient was hemodynamically stable, afebrile, and normotensive. Laboratory evaluations, including a complete blood count (CBC), magnesium, phosphorus, lactic acid, and comprehensive metabolic profile (CMP), were within normal limits, except for a mildly reduced estimated glomerular filtration rate (77 mL/minute/1.73 m²). A computed tomography (CT) scan of the abdomen and pelvis, performed with oral and intravenous contrast (Figure 1), suggested ileus or possible small bowel obstruction (SBO), with an intact distal colonic/rectal anastomosis and the presence of a small hiatal hernia. The patient was admitted under the care of the general surgery team, kept nil per os (NPO), and initiated on maintenance intravenous fluids (IVFs).

**Figure 2:**
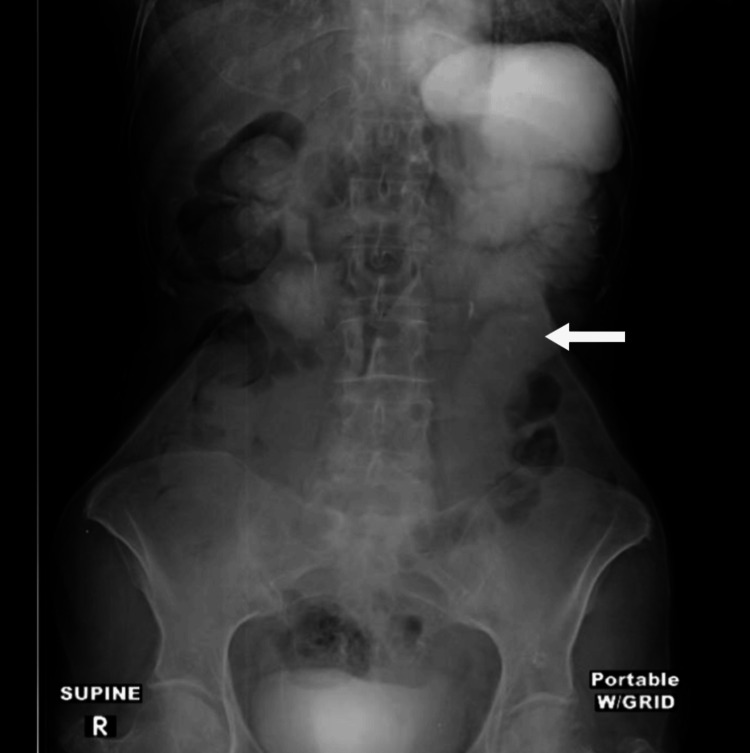
Abdominal X-Ray without contrast. The oral contrast from the previous day had only progressed into the proximal segments of the jejunum (white arrow), which were noted to be mildly distended.

**Figure 3:**
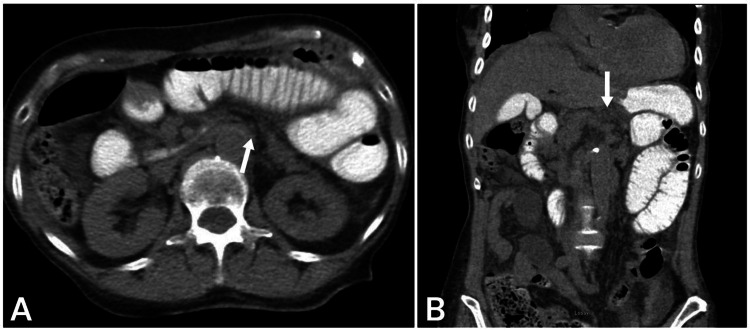
Non-contrast computed tomography (CT). Persistent small bowel distention, with residual contrast from prior imaging confined to the proximal small bowel. A subtle mesenteric rotation is noted in the mid-abdomen (white arrow). (a) Axial view and (b) coronal view.

On hospital day 2, the patient reported improvement in abdominal pain following the administration of analgesics. However, a follow-up abdominal X-ray (Figure 2) indicated that the oral contrast from a previous CT scan had only progressed into the proximal segments of the jejunum, which were mildly distended. On hospital day 3, despite being kept NPO with IVFs, the patient experienced worsening abdominal pain, nausea, and new-onset high-volume emesis. A repeat non-contrast CT scan (Figure 3) revealed persistent small bowel distention, with the contrast limited to the proximal small bowel and a slight mesenteric rotation in the mid-abdomen, raising concerns for an internal hernia. Conservative management was continued with close monitoring, and exploratory laparoscopy was considered if there was no improvement.

On hospital day 4, the patient's abdominal pain remained unchanged, leading to the insertion of a nasogastric (NG) tube to manage the emesis. By hospital day 5, the patient reported some relief in abdominal pain and nausea, with an abdominal X-ray revealing no distended loops of the bowel and a correctly positioned NG tube. However, on hospital day 6, the patient experienced a recurrence of worsening abdominal pain and distention. Due to the failure of conservative management, an urgent diagnostic laparoscopy was performed. Intraoperatively, a defect, likely iatrogenic, was identified in the transverse colon mesentery, with a segment of the small bowel completely herniated into the lesser sac and posterior to the stomach. The herniated bowel was successfully reduced, and the mesenteric defect was repaired with a V-Loc™ Wound Closure Device (Medtronic Minimally Invasive Therapies, Minneapolis, MN). The entire small bowel was inspected, showing no signs of ischemia, and appeared viable. On postoperative day 1 (hospital day 7), the patient reported no abdominal or incision site pain. Her diet was gradually advanced, which she tolerated well, and she experienced a return of bowel function. The patient was subsequently discharged home the following day in stable condition. During her postoperative visit two weeks later, she remained symptom-free and was healing well without further complications.

## Discussion

There are three main subtypes of transmesenteric hernias (THs): through a small bowel mesenteric defect, transmesocolic, and Petersen's [4]. TH is the most common type of hernia in the pediatric population, accounting for 30% of all IHs in this age group [2,4]. TH in this group is thought to be due to a congenital defect in the mesentery near the ileocecal region or the ligament of Treitz [4,5]. TH was once thought to be rare in the adult population; however, recent reports have shown that this is not the case [2,6]. In the adult population, THs may be due to iatrogenic causes, trauma, inflammation, and, most commonly, a previous history of abdominal surgery [2-4]. In our case, the probable cause of the transverse mesocolic internal hernia was the patient's recent Hartmann reversal procedure, which may have introduced a defect in the transverse mesocolon. Additionally, liver transplantation and Roux-en-Y bariatric surgery have been shown to be a risk factor for TH [2-4]. Both procedures involve the creation of a Roux-en-Y loop at the choledochojejunostomy site. If the loop is placed anteriorly to the transverse colon, referred to as antecolic, no defect will be created in the transverse mesocolon. This approach is not frequently used as a longer Roux loop is required. The most frequently used approach involves the creation of a defect in the transverse mesocolon. This allows a shorter loop and is referred to as a retrocolic approach [2-4]. With the rise in bariatric surgery and the frequent use of the retrocolic approach, the incidence of TH is expected to rise [2,7].

Patients typically present with features of intermittent or acute bowel obstruction, as in our patient listed above. The clinical manifestation may range from mild digestive symptoms to acute abdomen, including abdominal pain, intermittent or persistent, nausea, constipation or obstination, and abdominal distention [2,8]. Patients with complete SBO may present with more severe symptoms such as fever, tachycardia, hypotension and peritonitis, rebound, and guarding [2]. On a physical examination, a palpable mass may be felt, with abdominal tenderness [2].

The diagnosis of THs can often be difficult due to the wide range of presenting symptoms, their appearance, and variable locations in imaging studies [2]. The most common location is the right mid-abdomen adjacent to the abdominal wall [2]. This is probably due to the lack of a confining sac; therefore, the hernia may be located anywhere in the abdomen [8]. CT imaging is the gold standard for the diagnosis of internal hernias [9]. CT findings of a mesocolic internal hernia may show clustered small bowel loops near the abdominal wall, displaced mesenteric vessels, and the abnormal encapsulation of intestinal loops [8,9]. The herniated bowel is often lateral to the colon, with the transverse colon displaced centrally and inferiorly [9]. Key predictors include mesenteric vessel crowding, the displacement of the mesenteric trunk, and signs of small bowel obstruction, such as distension and the lack of contrast progression [2,9,10]. In our patient, a definitive diagnosis of TH was only made after exploratory laparotomy. As mentioned above, imaging studies elucidated some type of bowel obstruction; however, the exact cause was unknown.

The treatment of transverse mesocolic internal hernia may be conservative; surgical management may often be required as in the case of our patient [9]. For complete bowel obstruction and in the case of strangulation, swift operative management is required [9]. In the case of our patient, there were no clear indications of the presence of an internal hernia leading to bowel strangulation. The most common cause for SBO is the development of postsurgical adhesions, which can be managed conservatively [11]. This case exemplifies the diagnostic challenges of recognizing an internal hernia and whether the determination of such a hernia should have changed the management decisions.

## Conclusions

This case report emphasizes the need to consider internal hernias, particularly transverse mesocolic hernias, in patients with small bowel obstruction after recent abdominal surgery. Our 75-year-old patient's case highlights the diagnostic challenges and imaging limitations of this rare condition. Prompt surgical intervention is crucial to prevent complications such as bowel ischemia and strangulation. Raising awareness of internal hernias can improve early recognition and patient outcomes.
